# Supplementary material for: Membrane electrical properties of mouse hippocampal CA1 pyramidal neurons during strong inputs
Source: Biophys J. 2022 Jan 6;121(4):644–57. doi: 10.1016/j.bpj.2022.01.002 (PMC8873947; doi:10.1016/j.bpj.2022.01.002)
Supplement: Document S1. Supporting material [file mmc1.pdf]

Biophysical Journal, Volume *121*

## Supplemental information

# Membrane electrical properties of mouse hippocampal CA1 pyramidal neurons during strong inputs

**Daniela Bianchi, Rosanna Migliore, Paola Vitale, Machhindra Garad, Paula A. Pousinha, Helene Marie, Volkmar Lessmann, and Michele Migliore**

# Supporting Material

**Membrane electrical properties of mouse hippocampal CA1 pyramidal neurons during strong inputs**

D. Bianchi[1†], R. Migliore[1†], P. Vitale[1], M. Garad[2], P.A. Pousinha[3], H. Marie[3], V. Lessmann[2], M. Migliore[1*]

[1]Institute of Biophysics, National Research Council, Palermo, Italy

[2]Otto-von-Guericke University, Magdeburg, Germany

[3]Université Côte d'Azur, CNRS, IPMC, France

## 1. The $Na^+$ channel's model

In this section we describe in detail the kinetic of the transient $Na^+$ channel, used in this paper to reproduce the experimental findings, and implemented assuming that stimulus amplitude and duration generate a shift of the kinetics and reversal potential.

We started from a HH-like implementation of channel kinetics. The sodium current $i_{Na}$ was defined as

$$i_{Na}(t,v) = G_{Na}(t,v) \cdot (v - E_{Na}(t,v)),$$

where $v$ is the membrane potential, $E_{Na}$ the reversal potential, and $G_{Na}(t,v) = g_{Na} \cdot m^3(t,v) \cdot h(t,v)$ describes the channel's conductance change. It depends on the peak conductance $g_{Na}$ and on the activation and inactivation variables $m(t,v)$ and $h(t,v)$, respectively.

In our model, the reversal potential and the shift of activation/inactivation kinetics, with respect to their original values, were modulated by the EMA's function $f_{vrun}(t,v)$ (see main text for its definition) as

$$E_{Na}(t,v) = E_{Na}(t=0) - \alpha_{ENa} \cdot f_{vrun}(t,v)$$

$$sh_{Na}(t,v) = sh_{Na}(t=0) - \alpha_{Na} \cdot f_{vrun}(t,v)$$

Following this modulation, the original equations for the activation and inactivation variables

$$\frac{dm(t,v)}{dt} = \frac{m_\infty(t,v) - m(t,v)}{\tau_m(t,v)}$$

$$\frac{dh(t,v)}{dt} = \frac{h_\infty(t,v) - \mathrm{h}(t,v)}{\tau_h(t,v)}$$

were modified as follows.

The functions $a_m(t,v)$ and $b_m(t,v)$, defining the activation gate variable

$$m_\infty(t,v) = \frac{a_m(t,v)}{a_m(t,v) + b_m(t,v)}$$

and its time constant

$$\tau_m(t,v) = \begin{cases} \dfrac{2}{a_m(t,v) + b_m(t,v)} & \text{if} \quad \dfrac{2}{a_m(t,v) + b_m(t,v)} > 0.02 \\ else & 0.02\, ms \end{cases}$$

were modified as

$$a_m(t,v) = \begin{cases} \dfrac{0.4 \cdot \left[v - \left(v_m^{1/2} + sh_{Na}(t,v)\right)\right]}{1 - e^{-\frac{v - \left(v_m^{1/2} + sh_{Na}(t,v)\right)}{qa}}} & if\ \left|v - v_m^{1/2}\right| > 10^{-6} mV \\ else & 0.4 \cdot qa \end{cases}$$

$$b_m(t,v) = \begin{cases} \dfrac{-0.124 \cdot \left[v - \left(v_m^{1/2} + sh_{Na}(t,v)\right)\right]}{1 - e^{\frac{v - \left(v_m^{1/2} + sh_{Na}(t,v)\right)}{qa}}} & if\ \left|v - v_m^{1/2}\right| > 10^{-6} mV \\ else & 0.124 \cdot qa \end{cases}$$

Similarly, for the inactivation gate variable

$$h_\infty(t,v) = \frac{1}{\left(1 + e^{(v - v_h^{1/2} - sh_{Na}(t,v))/qinf)}\right)}$$

$$\tau_h(t,v) = \begin{cases} \dfrac{2}{a_h(t,v) + b_h(t,v)} & \text{if} \quad \dfrac{2}{a_h(t,v) + b_h(t,v)} > 0.05 \\ else & 0.05\, ms \end{cases}$$

The functions were modified as

$$a_h(t,v) = \begin{cases} \dfrac{0.03 \cdot \left[v - \left(v_h^{1/2} + sh_{Na}(t,v)\right)\right]}{1 - e^{-\frac{v - \left(v_h^{1/2} + sh_{Na}(t,v)\right)}{qd}}} & if\ \left|v - v_h^{1/2}\right| > 10^{-6} mV \\ else & 0.03 \cdot qd \end{cases}$$

$$b_h(t,v) = \begin{cases} \dfrac{-0.01 \cdot \left[v - \left(v_h^{\frac{1}{2}} + sh_{Na}(t,v)\right)\right]}{1 - e^{\frac{v - \left(v_h^{\frac{1}{2}} + sh_{Na}(t,v)\right)}{qg}}} & if\ \left|v - v_h^{\frac{1}{2}}\right| > 10^{-6} mV \\ else & 0.01 \cdot qg \end{cases}$$

with $qa = 7.2\,mV$, $v_m^{1/2} = -27\,mV$, $qinf = 2\,mV$, $v_h^{1/2} = -45\,mV$, $qd = 1.5\,mV$, $qg = 1.5\,mV$.

An analogous implementation of the dynamic shift was applied to potassium and calcium ionic currents.

## 2. The persistent $Na^+$ channel model

In this section we explicitly describe the equations governing the $Na^+$ persistent current used for Fig.5. The sodium current $i_{NaP}$ was defined as

$$i_{NaP}(t,v) = G_{NaP}(t,v) \cdot (v - E_{Na}),$$

where

$$G_{NaP}(t,v) = g_{NaP} \cdot m(t,v) \cdot h(t,v),$$

$E_{Na}$ is the reversal potential, $g_{NaP}$ the peak conductance, and $m(t,v)$ and $h(t,v)$ the activation and inactivation variables, described by the equations:

$$\frac{dm(t,v)}{dt} = \frac{m_\infty(t,v) - m(t,v)}{\tau_m}$$

and

$$\frac{dh(t,v)}{dt} = \frac{h_\infty(t,v) - \mathrm{h}(t,v)}{\tau_h}$$

where

$$m_\infty(t,v) = \frac{1}{[1 + \exp(-(v + 52.3)/6.8)]}$$

$$h_\infty(t,v) = \frac{1}{[1 + \exp((v + 48)/10)]}$$

with $\tau_h = 4000\,ms$, and $\tau_m = 1$ if $(m < m_\infty)$, else $\tau_m = 300$.

Simulation and model files are available for download at http://senselab.med.yale.edu/ModelDB/ acc.n. 266900.
