# Supplementary material for: Membrane electrical properties of mouse hippocampal CA1 pyramidal neurons during strong inputs
Source: Biophys J. 2022 Jan 6;121(4):644–57. doi: 10.1016/j.bpj.2022.01.002 (PMC8873947; doi:10.1016/j.bpj.2022.01.002)
Supplement: Document S2. Article plus supporting material [file mmc2.pdf]

Biophysical Journal
Article

Biophysical Society

Membrane electrical properties of mouse hippocampal CA1 pyramidal neurons during strong inputs

Daniela Bianchi,[1,5] Rosanna Migliore,[1,5] Paola Vitale,[1] Machhindra Garad,[2] Paula A. Pousinha,[3] Helene Marie,[3] Volkmar Lessmann,[2,4] and Michele Migliore[1,*]

[1]Institute of Biophysics, National Research Council, Palermo, Italy; [2]Otto-von-Guericke University, Magdeburg, Germany; [3]Université Côte d'Azur, CNRS, IPMC, Valbonne, France; and [4]Center for Behavioral Brain Sciences (CBBS), Magdeburg, Germany

ABSTRACT In this work, we highlight an electrophysiological feature often observed in recordings from mouse CA1 pyramidal cells that has so far been ignored by experimentalists and modelers. It consists of a large and dynamic increase in the depolarization baseline (i.e., the minimum value of the membrane potential between successive action potentials during a sustained input) in response to strong somatic current injections. Such an increase can directly affect neurotransmitter release properties and, more generally, the efficacy of synaptic transmission. However, it cannot be explained by any currently available conductance-based computational model. Here we present a model addressing this issue, demonstrating that experimental recordings can be reproduced by assuming that an input current modifies, in a time-dependent manner, the electrical and permeability properties of the neuron membrane by shifting the ionic reversal potentials and channel kinetics. For this reason, we propose that any detailed model of ion channel kinetics for neurons exhibiting this characteristic should be adapted to correctly represent the response and the synaptic integration process during strong and sustained inputs.

SIGNIFICANCE Stimulus-induced dynamic changes in neuron excitability, such as an increase in the spike threshold and of the depolarization baseline often observed in mouse recordings as a function of the current injection, are poorly understood. Conventional conductance-based computational models cannot reproduce these processes, and technical problems when measuring fast transient effects during a stimulus prevent adequate experimental investigation. Here we introduce a dynamic model suggesting that these effects may be caused by a significant dynamic alteration of the local membrane's ionic permeability.

INTRODUCTION

The standard experimental current-clamp protocols, used routinely and almost exclusively to establish the electrophysiological properties and the excitability profile of neurons, are to inject different currents into the soma and record its membrane voltage for a few hundreds of milliseconds. Different variations on this common theme are applied to study specific characteristics or properties. For example, a large and diversified set of injection protocols has been suggested to maximize the information that can be extracted from the recordings (1), and a systematic increase in stimulation frequency during a long current injection, called a zap current, is used widely to study resonance properties (2–4). Essentially the same protocols are used for neurons from any species, from squids to humans. Modelers have been using electrophysiological features extracted from traces recorded with these protocols to implement computational models able to reproduce and predict experimental findings regarding the response of a variety of neurons in different brain areas and under different conditions (5–7). Here we introduce and investigate a new electrophysiological feature often observed experimentally in mice. It consists of a large and transient increase in the average depolarization baseline (DBL), defined as the minimum value of the membrane potential between successive action potentials during a sustained input, as a function of current injection. This dynamic is inconsistent with the conventional Hodgkin-Huxley (HH) channel dynamics, and it cannot be reproduced by any known

Submitted June 8, 2021, and accepted for publication January 5, 2022.

[5]These authors contributed equally

*Correspondence: michele.migliore@cnr.it

Editor: Meyer Jackson.

https://doi.org/10.1016/j.bpj.2022.01.002

© 2022 Biophysical Society.

This is an open access article under the CC BY license (http://creativecommons.org/licenses/by/4.0/).

computational model. However, we show that it can significantly change a neuron's response to strong inputs in a way that can have important consequences for synaptic integration and transmission. So far, this DBL phenomenon has been completely neglected. Here we present a model that addresses this and suggest that it can be caused by a previously unnoticed and uncharacterized large dynamic change in the local cell's ionic permeability.

MATERIALS AND METHODS

Experiments

Laboratory 1

3- to 10-month-old C57BL6J/Rj mice (bred in house) were used. The animals were group housed in Makrolon cages at a temperature of 21°C ± 2°C and a 12:12 h light/dark cycle with lights on at 07:00 am. They had free access to food and water. All experimental procedures were performed in accordance with the ethical guidelines for the use of animals in experiments and in accordance with European Committees Council Directive 2010/63/EU.

The animal was anesthetized using ketamine (150 mg/kg)/xylazine (10 mg/kg) solution (Sigma-Aldrich, Lyon, France). Loss of consciousness was confirmed by absence of reflex activity following a toe pinch. The mouse was submitted to transcardial perfusion with an ice-cold sucrose cutting solution containing 2.5 mM KCl, 1.25 mM NaH_2PO_4, 10 mM $MgSO_4$, 0.5 mM $CaCl_2$, 11 mM glucose, 234 mM sucrose, and 26 mM $NaHCO_3$, saturated with 95% O_2 and 5% CO_2. The mouse was then decapitated immediately, the brain was removed, and the hippocampus was dissected and left to cool down in ice-cold sucrose cutting solution for 5 min. The hippocampus was then mounted in an agar support and placed in oxygenated ice-cold sucrose cutting solution. 350-μm-thick transverse hippocampal slices were collected, starting from the first slice that allowed identification of a clearly separated dentate gyrus and all *cornu ammonis* (CA) regions using a vibratome (Microm HM600V, Thermo Scientific, France). Slices were then placed in a pre-warmed chamber containing artificial cerebrospinal fluid (aCSF) composed of 119 mM NaCl, 2.5 mM KCl, 1.25 mM NaH_2PO_4, 1.3 mM $MgSO_4$, 2.5 mM $CaCl_2$, 26 mM $NaHCO_3$, and 11 mM glucose (pH 7.4, 290–295 mOsm/L) saturated with 95% O_2 and 5% CO_2, placed in a water bath at 37°C for 1 h, and then kept at room temperature until use.

For whole-cell recordings, slices were transferred to a recording chamber containing continuously circulating (2 mL/min) oxygenated and warm (31°C–33°C) aCSF on an upright microscope with infrared differential interference contrast (IR-DIC) illumination and epifluorescence (Scientifica, UK). Pyramidal neurons in the CA1 region of the hippocampus were visualized with differential interference contrast infrared video microscopy (WAT-902H ultimate camera [Watec, France] coupled to Patchvision software [Scientifica]). A tight seal (>1 GΩ) on the cell body of CA1 neurons was obtained with fire-polished glass pipettes (pipette resistance, 4–6 MΩ) filled with intracellular solution containing 135 mM K-D-gluconate, 5 mM NaCl, 2 mM $MgCl_2$, 10 mM HEPES, 0.5 mM EGTA, 2 mM MgATP, and 0.4 mM NaGTP; pH was adjusted to 7.25 using 1 M KOH (285–290 mOsm/L). The resting membrane potential was first measured in the absence of any spontaneous firing, and only cells more negative than −60 mV at the start of recording were considered for further analysis. Cells selected for recordings had an average input resistance of 141 ± 60.6 MΩ (standard deviation, $n = 32$). The intrinsic excitability of CA1 pyramidal neurons was assessed in current-clamp mode. Pipette capacitance, CA1 pyramidal cell capacitance, and serial resistance were compensated manually. Their values were not recorded. At the beginning of an experiment, the membrane potential of the neuron was set to a value close to −65 mV (average value of 64.4 ± 5.5 mV, $n = 32$) using holding currents between −100 and +75 pA. The action potential frequency readout was then obtained in response to 0–400 pA (50-pA increments) depolarizing somatic current injections of 400-ms duration. Traces reported in the figures include a −15-mV liquid junction potential correction. Recordings were obtained using a Multiclamp 700B (Molecular Devices, Sunnyvale, CA, USA). Signals were collected and stored using a Digidata 1440 A converter and pCLAMP 10.2 software (Molecular Devices). Data were filtered at 2 kHz and digitized at 10 kHz. In total, 226 traces from 32 cells were used for this work.

Laboratory 2

Six-month-old C57BL6J/Rj mice, derived from our own breeding colony, were used. The animals were group housed in Makrolon cages at a temperature of 21°C ± 2°C and a 12:12 h light/dark cycle with lights on at 07:00 am. They had free access to food and water. All experimental procedures were performed in accordance with the ethical guidelines for the use of animals in experiments and in accordance with European Committees Council Directive 2010/63/EU. All experimental procedures were approved by the Landesverwaltungsamt Saxony-Anhalt.

The animal was anesthetized using isoflurane (Isofluran CP, cp-pharma, Germany). Loss of consciousness was confirmed by absence of reflex activity following a toe pinch. The mouse was decapitated immediately, and the brain was separated from the skull. The brain was kept in ice-cold aCSF containing 125 mM NaCl, 2.5 mM KCl, 25 mM $NaHCO_3$, 0.8 mM NaH_2PO_4, 25 mM glucose, 1 mM $MgCl_2$, and 2 mM $CaCl_2$ saturated with 95% O_2 and 5% CO_2 (pH 7.2–7.4, 301–304 mOsm/L, Fiske Micro-osmometer Model 210, Fiske Associates, USA). The cerebellum, brain stem, and one-third of the frontal brain were removed before brain slicing. The ventral part of the brain was cut transversely at an angle of 11° to obtain transversal slices. The brain was cut with a vibratome (VT1200S vibratome, Leica Biosystems, Germany), and 350-μm-thick acute hippocampal slices were collected, starting from the first slice that allowed identification of a clearly separated dentate gyrus and all CA regions. These slices were used for intrinsic excitability experiments and represent the intermediate and ventral region of the hippocampus. About 6 slices were transferred into the interface-style chamber and allowed to incubate for 25 min in continuously carboxygenated (5% CO_2, 95% O_2), pre-warmed aCSF (200 mL, same composition as the slice preparation medium mentioned above) at 34°C–35°C to allow the slice surface to recover from blade trauma, followed by at least 1 h of recovery at room temperature. All slices were maintained in this interface chamber at room temperature until transfer to the recording chamber of an upright microscope for electrophysiological recordings.

For all experiments, 350-μm-thick acute, transversal hippocampal slices were used. For whole-cell recordings, pyramidal neurons in the CA1 region of hippocampus were visualized with differential interference contrast infrared video microscopy (VX45 camera, Optronis, Germany; Examiner A1 microscope, Zeiss). aCSF was composed of 125 mM NaCl, 2.5 mM KCl, 25 mM $NaHCO_3$, 0.8 NaH_2PO_4, 25 glucose, 2 mM $CaCl_2$, and 1 mM $MgCl_2$ saturated with 95% O_2 and 5% CO_2 (pH 7.2–7.4, 301–304 mOsm/L). Slices were incubated for 5–10 min in the recording chamber before start of recording. Whole-cell recordings were performed at 34°C–36°C with glass pipettes (pipette resistance, 4–6 MΩ) filled with intracellular solution containing 140 mM potassium gluconate, 10 mM HEPES, 20 mM KCl, 4 mM Mg-ATP, 0.3 mM Na-GTP, and 10 mM Na-phosphocreatine; pH was adjusted to 7.2–7.4 using 1 M KOH (280–290 mOsm/L). Pipette capacitance, CA1 pyramidal cell capacitance, and serial resistance were manually compensated with an EPC8 patch-clamp amplifier. Their values were not recorded. The intrinsic excitability of CA1 pyramidal neurons was assessed by action potential frequency readout in response to 0–450 pA (50-pA increments, repeated five times for each current injection at 5-s intervals) depolarizing somatic current injections for 500-ms duration in current-clamp mode from the endogenous resting membrane potential (i.e., no holding current was used). Cells selected for recordings had an average input resistance of 141.6 ± 25.95 MΩ ($n = 35$), and a resting membrane potential of 67.4 ± 3.9 mV (range, −74 to −62 mV). All traces

reported in all figures include a −10-mV liquid junction potential correction. Cells with an endogenous resting membrane potential more positive than −60 mV at the start of the recording were discarded from analysis. Recordings were performed using an EPC8 patch-clamp amplifier connected to a LiH8+8 interface (HEKA, Germany) and acquired with Patchmaster software (HEKA). Data were filtered at 3 kHz and digitized at 10 kHz. Data analyses were performed using FitMaster (HEKA). In total, 350 traces from 7 cells were used for this work.

Modeling

All simulations were carried out using the NEURON simulation environment (8). In a few cases we used NEURON integrated with Python (9) into a parallel code executed on different high-performance computing systems: JURECA (Juelich Supercomputing Center, Germany), Galileo (CINECA, Italy), Piz Daint (Swiss National Supercomputing Center CSCS), and the Neuroscience Gateway (San Diego, CA, USA (10)). A morphology reconstruction of a CA1 pyramidal neuron from the same strain used in the experiments was downloaded from http://www.neuromorpho.org (cell fx_CA1_8.CNG.swc). The channel kinetics were based on those used in previously published papers on CA1 hippocampal neurons and validated against a number of experimental findings. In particular, the model was implemented by merging model files from Migliore et al. (11) (ModelDB account number 244688) and Bianchi et al. (12) (ModelDB account number 143719). From Migliore et al. (11), we used a delayed-rectifier type currents (K_{DR}), two A-type potassium (K_A) currents (for proximal and distal dendrites), a non-specific hyperpolarization-activated current (I_h), three types of voltage-dependent calcium currents (CaL, CaT, and CaN), and two types of calcium-dependent K^+ current (a slow and a medium afterhyperpolarization (AHP) current). The Na^+ and R-type Ca^{2+} current, M-type potassium current, and the calcium pump were taken from Bianchi et al. (12). The calcium currents were distributed according to experimental findings (13), and the K_A and I_h increased linearly with distance from the soma (14,15). Passive properties and peak conductance for each channel were adapted from their original values to qualitatively reproduce the specific experimental findings used in this work. For simulations using synaptic inputs, we implemented two main excitatory synaptic afferent pathways as in (16), following experimental findings (17): one mimicking entorhinal cortex (EC) inputs (40 synapses based on α-amino-3-hydroxy-5-methyl-4-isoxazolepropionic acid receptors (AMPA), each with a peak conductance of 0.6 nS), randomly located in distal dendrites (>140 μm from the soma), and another one modeling CA3 Schaffer collateral (SC) inputs (20 AMPA and 20 synapses based on N-methyl D-aspartate receptors (NMDA) with maximum peak conductances of 0.5 and 0.25 nS, respectively) randomly located in the proximal dendrites. Synaptic activation details are discussed in Response to synaptic inputs. Simulation and model files are available on ModelDB (http://senselab.med.yale.edu/ModelDB/, account number 266900) and as an interactive entry in the "Live Papers" section of the Cellular Level Simulation Platform of the EBRAINS Infrastructure (https://humanbrainproject.github.io/hbp-bsp-live-papers). For each trace, the DBL was calculated as the average value of the minimum voltage between spikes for all spikes in the train.

RESULTS

Experimental traces exhibit a large increase of DBL with current injection

Fig. 1 illustrates the new electrophysiological feature in a few typical experimental recordings. Here we show the somatic membrane potential from four CA1 pyramidal neurons under increasing somatic current injections. They were obtained independently in two labs (laboratory 1 and laboratory 2 in Fig. 1) from mice of the same strain under almost identical recording conditions and show some variability in terms of spike number, amplitude, and overall pattern (e.g., compare the traces for the two cells from laboratory 1). This can be expected because, as noted previously for rats (11), recordings from different neurons belonging to the same population exhibit a large variability of their response to a given external input. However, in contrast with the recordings obtained from rats, in mice, the DBL increased systematically with increasing current injection for all CA1 neurons recorded. DBL values, its progression with increasing current injections, and the maximum value (indicated by a dashed line in Fig. 1) were different in different neurons. It is important to note that this increase was also associated with an increase in the spike threshold. Furthermore, the peak amplitude of the action potentials (APs) was not changed significantly (Mann-Whitney test, $p = 0.244$, calculated from the first AP of traces for 0.15- and 0.4-nA current injection). This is surprising, given that the increase of the DBL should result in incomplete Na^+ current recovery from inactivation. These results suggest that pyramidal neurons of mice, at least in the CA1 region of the hippocampus, may harbor an intrinsic electrophysiological mechanism responsible for limiting membrane repolarization after an AP, during a sustained input, without affecting the peak AP amplitude. In neurons where the DBL is higher than the threshold for synaptic release, this mechanism may significantly modulate network activity in a way that has so far not been considered.

The DBL level can be different in different neurons and is associated with an increase in the spike threshold as a function of the input

A more detailed illustration of the DBL experimental variability is shown in Fig. 2 *A*, where we plot the DBL as a function of the somatic current injection for all cells used in this work, together with the average values calculated from the results of each laboratory. We found that DBL levels were spread over a wide range of values, with the somatic potential being depolarized up to +60 mV from the resting potential. This is a huge effect that may strongly affect the input/output (I/O) properties of a neuron. A large variability within and between laboratories is evident, with many neurons from both laboratories exhibiting a similar DBL profile (overlapping red and blues lines in Fig. 2 *A*). It should be noted that, even when all traces start from a similar resting potential (approximately −80/−70 mV; Fig. 1), because of the slightly different experimental protocol used in the two laboratories, we found that groups of neurons from different laboratories exhibited average values of DBL that are significantly different (Mann-Whitney or *t*-test, $p < 0.001$ for all currents). This difference may be

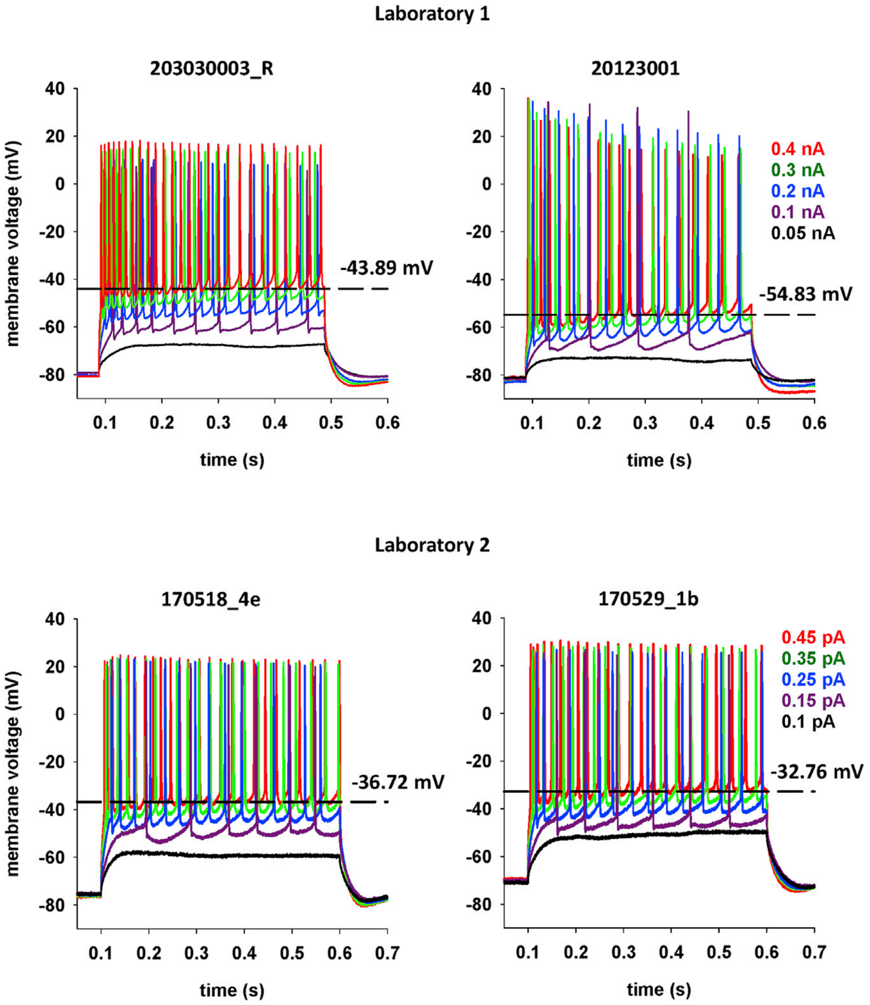

FIGURE 1 DBL increases with current injection. Typical somatic membrane potential from four cells from two laboratories, in response to 400 (top) or 500 ms (bottom) depolarizing current steps of different amplitude injected into the soma. The dashed lines mark the highest DBL value observed in each neuron..

caused by the animal's age, experimental procedures, the specific recording location, or some other morphological, electrochemical, or physiological condition. The investigation of this issue was outside the scope of this paper, but, nonetheless, these results demonstrated that the DBL is a clear and measurable effect and that it can be observed in traces recorded in independent laboratories. A clear input current-dependent change was also observed for the spike

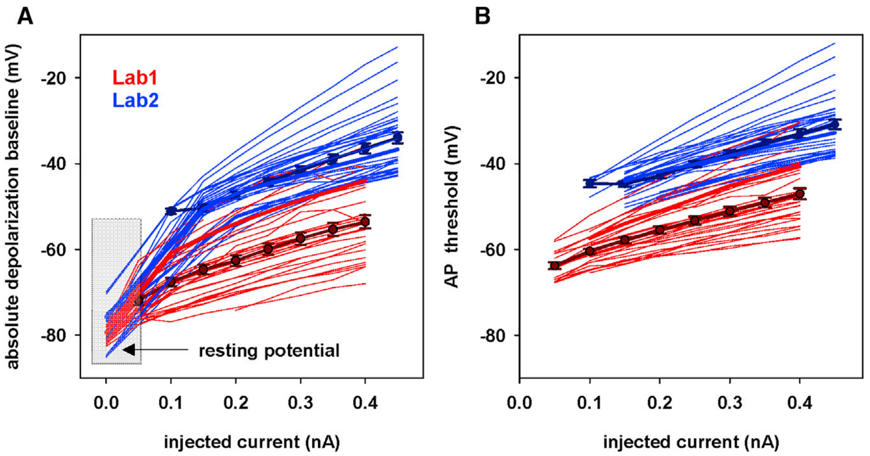

FIGURE 2 The DBL and spike threshold can be very different in different neurons. (*A*) Thick lines and symbols represent average (±standard error (SE)) DBL values, calculated from all cells from the two laboratories, in response to 400-ms (laboratory 1) or 500-ms (laboratory 2) depolarizing current steps. Thick red and blue lines represent the DBL of cell 203030003_R (from laboratory 1) and cell 170518_4e (from laboratory 2), respectively. Thin lines represent results for all the other cells. (*B*) Average spike threshold, automatically calculated from all APs in a train with the feature extraction tool available on the EBRAINS Platform of the Human Brain Project (https://hbp-bsp-hhnb.cineca.it/efelg/). Thick lines and symbols represent average (±SE) values calculated from all cells from the two laboratories; thick red and blue lines represent cell 203030003_R and cell 170518_4e, respectively; and thin lines indicate results for all other cells.

threshold (Fig. 2 *B*). If, as we hypothesize, these effects are caused by one or more electrophysiological mechanisms that so far have not been considered, then it is important to have a model able to capture them to have a better representation of the intrinsic membrane properties. For these reason, we decided to further investigate the possible underlying mechanisms.

Conventional computational models cannot reproduce the observed change in the spike threshold

Our first step was to illustrate why a conventional model cannot reproduce the experimentally observed increase in the spike threshold. In the left panel of Fig. 3 *A* , we plot the Na^+ steady-state activation and inactivation curves, highlighting (Fig. 3 *A*, shaded area) the range of experimentally observed DBL, and the first two APs, from the same neuron, recorded during a 0.1- and 0.4-nA current step (Fig. 3 *A*, center panel). The spike threshold increase in this case was ~10 mV (dashed lines in Fig. 3 *A*, center panel), and the first two APs had a very similar amplitude and dynamics but shifted by approximately the same amount (Fig. 3 *A*, right panel). We then arranged a model using only a limited set of channels (Na, K_{DR}, and K_A) to simplify interpretation of the results, and their peak channel conductance was adjusted to qualitatively reproduce the first AP obtained experimentally for 0.1- and 0.4-nA injections (Fig. 3 *B*, left and center panels). A direct comparison between experimental and model traces at 0.4 nA (Fig. 3 *B*, center panel) highlights the problem. The model is not able to reproduce the higher DBL, the higher spike threshold, and the amplitude of the AP following the first one in the train; in contrast with the experimental trace, the membrane potential quickly enters a depolarization block state after the first AP. The dynamic of the underlying biophysical mechanism is illustrated in Fig. 3 *C*, where we plot the instantaneous value of the Na^+ activation and inactivation gate variables during the simulations with 0.1 and 0.4 nA. During each AP elicited by the weak 0.1-nA input, the Na^+ activation and inactivation gate variables (Fig. 3 *C*, left panel) follow a complete cycle that brings them close to their original steady-state value soon after the AP ends and the membrane repolarizes. This occurs even when the depolarizing effect of the sustained input current causes the membrane potential to return to approximately −60mV instead of its natural resting value of approximately −80 mV. The neuron is able to elicit additional APs at full amplitude because, in this range of membrane potential, the activation and inactivation gate variables can fully recover their initial state (see gray area in Fig. 3 *A*, left). Note, in particular, that the inactivation variable (gray line in Fig. 3 *C*, left) returns to a value close to 1 before the second spike. The situation during a stronger input is drastically different (Fig. 3 *C*, right). In this case, after the first spike, the input current drives the membrane into a more depolarized level, and this prevents full recovery from inactivation (gray line in Fig. 3 *C*, right). The end result is that there is not enough Na^+ current available to generate another full-amplitude AP, and the cell goes into a depolarization block

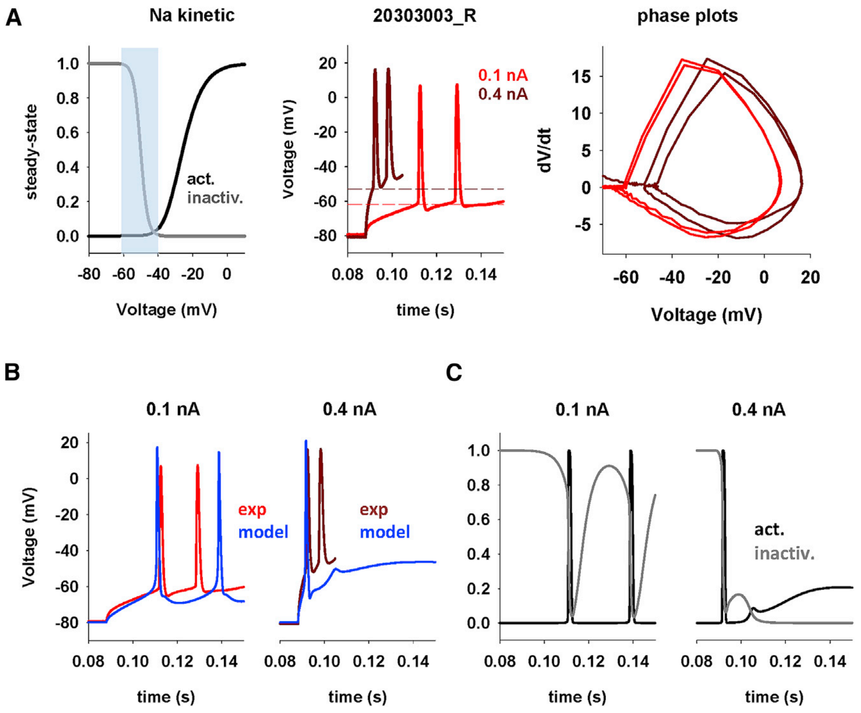

FIGURE 3 A conventional model cannot take into account the change in the spike threshold. (*A*) Steady-state Na^+ activation and inactivation kinetics; the gray area represents the range of experimentally observed DBL (left). Initial APs of cell 203030003_R in response to current injections of 0.1 and 0.4 nA; dashed lines represent the corresponding spike threshold values (center). Phase plots from experimental traces at 0.1 and 0.4 nA (right). (*B*) Somatic membrane potential from model and experiments for 0.1 nA and 0.4 nA somatic current injections. (*C*) Sodium activation and inactivation gate variables during current injections of 0.1 nA (left) and 0.4 nA (right).

state. This effect is not observed in the experimental recordings, where APs are elicited at full amplitude even for currents strengths that drive the membrane potential well above the range required for full recovery of Na^+ activation/inactivation. These results should make it clear why conventional conductance-based models cannot reproduce experimental findings. With a DBL above approximately −60 mV, there is no way for Na^+ channels to fully recover from inactivation after the first AP, and a sustained input will inevitably fail to elicit full-amplitude APs. It may be argued that, in principle, Na^+ channels with resurgent properties (18) could help because they can provide an additional depolarizing drive. However, the size of the resurgent component (approximately 10% of the main current) and its kinetic properties (fast activation soon after AP repolarization) makes them unsuitable to reproduce, even qualitatively, the transient increase in the spike threshold observed experimentally.

Biophysical mechanisms that can underlie the observed DBL level

To investigate the mechanisms that can be responsible for the DBL, we considered the experimental trace recorded from a neuron (cell 203030003_R) in response to a 0.3-nA somatic current injection compared with a conventional conductance-based model (Fig. 4 *A*, left). The number of APs was well reproduced by the model, but there were two strong discrepancies between the recording and the model trace: 1) the model was unable to reproduce the experimentally observed DBL at the beginning or the current step, and 2) the DBL decreased further during the train of APs, in striking contrast with experiments (red trace in Fig. 4 *A*, left).

To explore possible solutions for these problems, it is important to note that, although the DBL at the beginning of a current step depends on the dynamic interaction between the Na^+ and K^+ channels to elicit APs at full amplitude, its reduction during a train of APs mainly depends on progressive activation of the M-type potassium current and Ca-dependent K^+ current. The results presented in the previous section (Fig. 3) suggested that the only plausible biophysical mechanism able to reproduce full-amplitude APs during a sustained input is a transient and input-dependent shift of channels' kinetics. This is shown in Fig. 4 *A*, right panel, where we show how the model can reproduce the initial DBL by assuming a +11-mV shift of all channels' kinetics.

We now consider the progressive DBL reduction during a train. It may be argued that a transmembrane channel expressed specifically on the mouse neuron membrane, with peculiar kinetic properties, could contribute to maintaining a stable DBL during a train of APs. In principle, any relatively slow inward ionic current could provide a progressive depolarizing component that could compensate for the progressive opening of the outward K^+ currents during a sustained input. The non-specific I_h current, with a reversal potential around −30mV (15), or a persistent Na^+ current (19,20) appear to be suitable candidates for this. The non-specific I_h current cannot help because it inactivates with depolarization, and, in CA1 pyramidal neurons, it has an inhibitory effect (21). To test whether a persistent Na^+ current (Nap) can help to maintain a stable DBL during a train, we used a previously published kinetic model for CA1

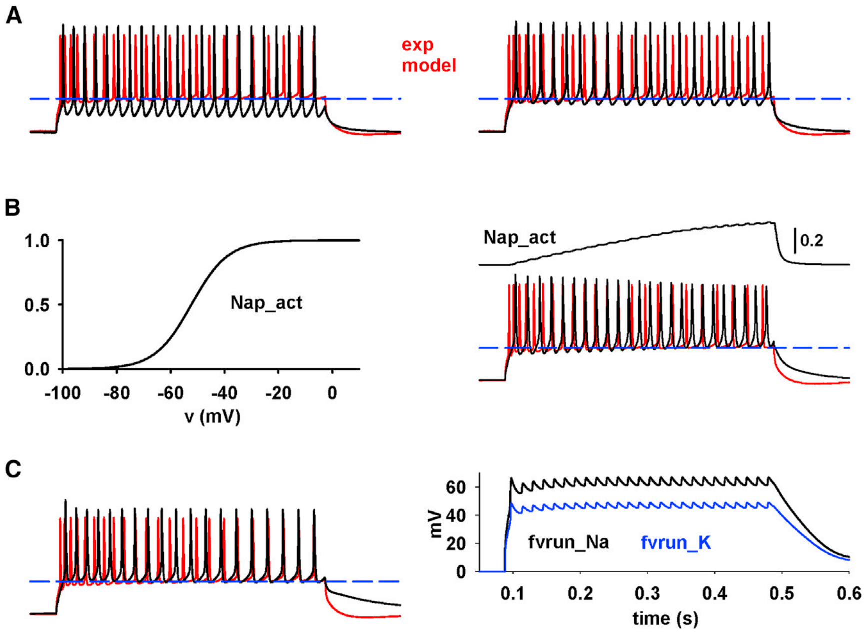

FIGURE 4 Modeling the mechanisms underlying a DBL. (*A*) Left: cell 203030003_R firing pattern in response to a 0.3-nA somatic current injection (red line) and modeling results using a conventional model for ion channel kinetics (black line). Right: comparison of experimental and model traces after a +11-mV shift of Na^+ and K^+ channel kinetics (black line). (*B*) Left: steady-state activation curve of the persistent Na^+ current (Nap). Right: comparison of experimental and model traces using a Nap; the top trace represents Nap activation during the simulation. (*C*) Left: comparison of the experimental trace with a simulation obtained using a dynamic shift of channel kinetics and reversal potential. Right: corresponding plots of the f_{vrun} function for Na^+ and K^+ during the simulation. Model traces were sampled at 10 KHz and filtered at 2 kHz, as in the experiments.

pyramidal neurons (ModelDB account number 123927 (20); Fig. 4 *B*, left). We found that, by modifying the activation and deactivation time constants to 300 ms and 1 ms, respectively, the DBL was in very good agreement with the experiment, as shown in Fig. 4 *B*, right panel. However, this mechanism has the major drawback of making the cell progressively more excitable than in the experiments, decreasing the interspike interval during the current step, in striking contrast with the experimental trace. Because the Nap is a depolarizing current, this effect cannot be avoided. Essentially the same effect would be generated by any other inward current, such as a calcium current.

We thus hypothesized that another possible mechanism would be an input-dependent change in membrane ionic permeability, which could transiently and significantly reduce the ionic chemical gradient across the membrane of a cell through a progressive shift of the ion reversal potentials. Together with the input-dependent shift in the channels' kinetics, these mechanisms could explain the DBL at all currents. To model these effects, we considered the instantaneous and past neuron activity to generate, during a simulation, a dynamic shift of the reversal potential, E_{ion}, and of the activation/inactivation kinetics (sh_{ion}) of the ionic currents, using an exponential moving average (EMA) (22,23) of the membrane potential, v.

An EMA is a type of Moving Average that places a greater weight and significance on the most recent data points.

The equations for EMA can be written as

$$EMA[1] = x[1]$$

$$EMA[n] = \alpha x[n] + (1-\alpha)EMA[n-1]$$

, where n is the number of data points, $EMA[n]$ the current output, $EMA[n-1]$ the previous output, and α a coefficient ($0<\alpha<1$) representing the amount of weight decrease. A higher α reduces the weight of older observations faster, and the factor for previous inputs decreases exponentially as

$$\begin{aligned} EMA[n] &= \alpha x[n] + (1-\alpha)EMA[n-1] = \\ &= \alpha x[n] + (1-\alpha)(\alpha x[n-1] + (1-\alpha)EMA[n-2]) = \\ &= \alpha x[n] + (1-\alpha)(\alpha x[n-1] + (1-\alpha)(\alpha x[n-2] \\ &\quad +(1-\alpha)(\alpha x[n-3] + (1-\alpha)EMA[n-3]) = \ldots \\ &\ldots = \alpha\sum_{k=1}^{n}(1-\alpha)^k x[n-k] \end{aligned}$$

; the weight of $x[n-i]$ is thus $\alpha(1-\alpha)^i$.

We applied EMA using

$$\alpha = \begin{cases} \dfrac{2}{n+1} & n = 2,\ldots,t_{step} \\ \dfrac{2}{t_{step}+1} & n>t_{step} \end{cases}$$

and calculated as

$$EMA[1] = x[1]\ ,$$

$$\begin{aligned} EMA[s] &= \frac{2}{s+1}x[s] + \left(1-\frac{2}{s+1}\right)\mathrm{EMA}[s-1] \\ &= \frac{2}{s+1}x[s] + \left(\frac{s-1}{s+1}\right)\mathrm{EMA}[s-1]\ , \end{aligned}$$

if $s = 2,\ldots,t_{step}$, or

$$\begin{aligned} EMA[n] &= \frac{2}{t_{step}+1}x[n] + \left(1-\frac{2}{t_{step}+1}\right)\mathrm{EMA}[n-1] \\ &= \frac{2}{t_{step}+1}\sum_{k=t_{step}}^{n}\left(1-\frac{2}{t_{step}+1}\right)^k x[n-k], \end{aligned}$$

for $n>t_{step}$.

In our case, $x = v - v_{rest}$, so we obtain

$$\begin{aligned} EMA(t,v) &= [x - EMA(t-1,v)]\bullet\frac{F_i}{cnt+1} + EMA(t-1,v) = \\ &= x\bullet\frac{F_i}{cnt+1} + \left(1-\frac{F_i}{cnt+1}\right)EMA(t-1,v) \end{aligned}$$

, where $cnt = \mathrm{t}\ if\ (t<t_{step}+1)\ \ else\ cnt = t_{step}$ and t_{step} is the number of simulation time steps within the fixed time window. With preliminary tests, we found that a $t_{step} = 1000$ was sufficient to appropriately consider the past activity history. The shift of E_{ion} and sh_{ion} was calculated from the EMA as

$$f_{vrun}(t,v) = B_iC_i\frac{EMA(t,v)}{C_i + EMA(t,v)}, \quad (1)$$

$$E_j(t,v) = E_j(t=0) - \alpha_{Ej}\bullet f_{vrun}(t,v),$$

$$sh_i(t,v) = sh_i(t=0) + \alpha_i\bullet f_{vrun}(t,v).$$

In these equations, v_{rest} is the resting membrane potential, cnt is the EMA time window size (in time step units) and f_{vrun} is a Hill function of the EMA, $F_i = 2$, and α_j, α_i, B_i and C_i parameters, with $j = \{Na,\ K\}$ and $i = \{Na,\ K,\ Ca\}$.

Because the amount of this effect is cell and input current specific, when modeling a specific neuron, the parameters

would need to be tuned in a neuron-by-neuron manner, just as the set of peak channel conductances. In Fig. 4 *C* we compare a simulation with the experimental trace for a 0.3-nA somatic current injection and the corresponding plots of the f_{vrun} function for Na^+ and K^+ during the simulation. These results suggest that, in mouse neurons, there are mechanisms in effect that could dynamically alter the electrophysiological properties of the membrane during sustained current injections.

Dynamic change in the cell's local ionic permeability explains the DBL increase

In the previous section we introduced an effective and biophysically plausible model able to reproduce the experimentally observed increase in DBL during a sustained input. It suggested that a current injection could transiently modulate channel kinetics and ion reversal potentials. Because virtually all conductance-based computational models assume these properties to be constant, it should be clear why they cannot reproduce the DBL dynamics. We tested the robustness of our model, using as a reference the entire experimental current sweep recordings from two neurons, one from each laboratory (cells 203030003_R and 170,518_ 4e; Fig. 1). A manual trial-and-error procedure was used independently for the two neurons to find a set of parameters able to simultaneously reproduce the firing pattern and the DBL observed experimentally for all current injections. During the fit procedure, we prioritized only the average spike frequency as a function of the current injection because this is by far the most important electrophysiological feature that needs to be reproduced for an individual neuron model, especially when it is meant to be integrated into a network. In this case, we preferred a manual rather than an automatic fitting procedure. The rationale for this choice is that automatic fitting is appropriate when a basic set of validated mechanisms is available. This is what we did, for example, when implementing a unified pipeline approach to generate families of biophysical models for rat CA1 pyramidal neurons and interneurons (11) or to find out how and to what extent specific biochemical pathways involved in synaptic transmission can be affected by pharmacological or genetic manipulation (24,25). In a case like the one presented in this paper, where no previous information is available regarding the underlying mechanisms, we believe that a manual procedure should be preferred because it allows better determination of qualitative correlations between parameters. The main point here was to determine whether the hypothesized mechanisms are able to explain the puzzling experimental findings. This will then set the stage for an automatic fitting procedure able to routinely find ensembles of parameters fitting equally well the DBL of any given neurons or neuron population. Typical results are shown in Fig. 5, where we compare model and experimental traces for 0.15 and 0.35 nA. The DBL-related parameters for each neuron are reported in Table 1.

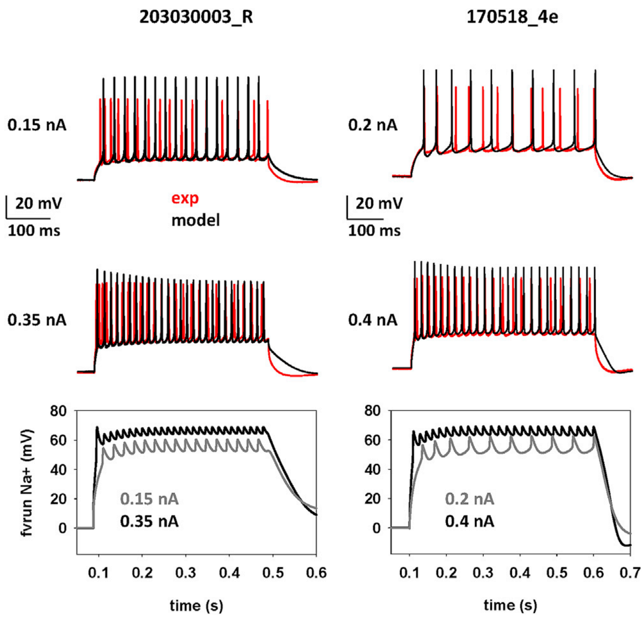

FIGURE 5 A dynamic shift of channel kinetics and reversal potential can take into account the DBL. Top: somatic membrane potential during different current injections in the model (black traces) and in experiments (red traces) for cell 203030003_R (left, from laboratory 1) and cell 170518_4e (right, from laboratory 2). Bottom: time evolution of the $f_{vrun}(t, v)$ function in the model of the two cells. Model traces were sampled at 10 KHz and filtered at 2 kHz (for cell 203030003_R) and 3 KHz (for cell 170518_4e), as in the experiments.

The model was in very good agreement with the experiments in terms of number of spikes and extent of DBL. These results suggest that the new model introduced here is able to take into account the dynamic change of the electric properties of a neuron during sustained inputs.

Next we carried out a more systematic comparison between experiments and models. The results are shown in Fig. 6, where we report, for the two neurons, the average spike frequency and DBL as a function of the injected current. The model was able to also capture the correct trend for the DBL level as a function of the input current (black plots in Fig. 6), with a deviation from experimental values that was never higher than a few millivolts. A conventional model was clearly not able to reproduce the DBL level (blue plots in Fig. 6). The different experimental range of DBL level observed between the two cells can be correlated to their different firing frequencies and suggests that morphological characteristics may also underlie the level of DBL observed from the soma of any given cell. We did not investigate this issue further because it would have required a detailed reconstruction of the very same cells from which the recordings were obtained. These results suggest that our approach is an effective way to reproduce the experimentally observed DBL in mouse hippocampal pyramidal neurons.

TABLE 1 f_{vrun} **Parameters**

Parameter	α_{ENa}	α_{Na}	B_{Na}	C_{Na}	α_{EK}	α_{K}	B_{K}	C_{K}	α_{Ca}	B_{Ca}	C_{Ca}
203030003_R	0.25	0.13	2.60	60	1.06	0.15	2.11	48	0.15	2	50
170518_4e	0.30	0.17	2.50	68	1.12	0.12	2.13	68	0.10	2	70

Values are for cell 203030003_R (from Laboratory 1) and cell 170518_4e (from Laboratory 2).

Predicted shifts of channel kinetics and ion reversal potentials

The shift our model predicted to reproduce the experimental recordings, as a function of the input current, is shown for the two neurons in Fig. 7, left panels, for the Na^+ and the K^+ reversal potential (the Ca^{2+} reversal potential is updated explicitly during the simulation) and in Fig. 7, right panels, for the shift of the activation/inactivation kinetics. The change predicted in the two neurons for the reversal potential of Na^+ was between 5 and 20 mV, similar for the two neurons, whereas a much larger change, between 15 and 65 mV, was required for the K^+ current, with a range that was different for the two neurons (approximately 30–50 mV for neuron 203030003_R and 20–65 mV for neuron 170518_4e). For channel kinetics, the model predicted a shift of up to approximately 10 mV for all channels, with the Na^+ channel requiring a larger shift with respect to K^+ and Ca^{2+} channels. These results suggest that the experimentally observed DBL can be caused by a cell-dependent transient alteration of channel kinetics and ion current reversal potential in response to external inputs.

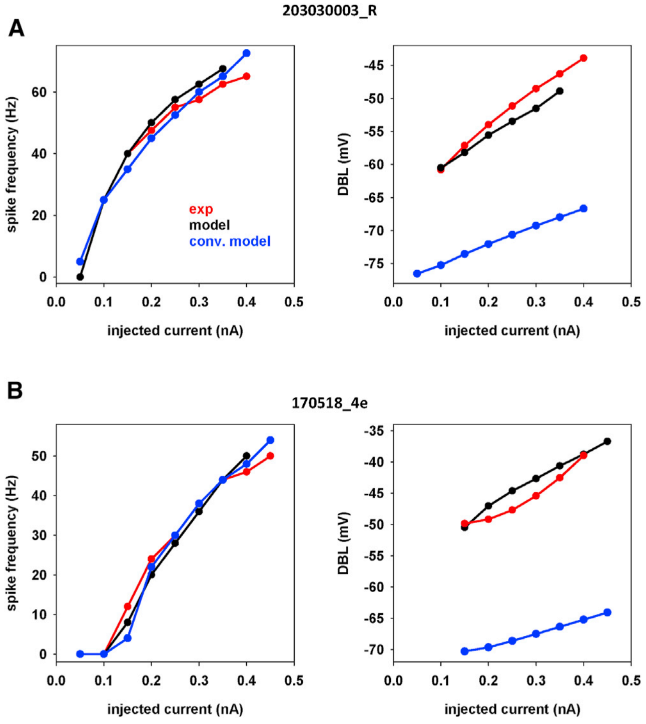

FIGURE 6 Comparison of results obtained for spike frequency and DBL level as a function of current injection, using a conventional HH-like model (blue), our model (black), and experiments (red). (*A*) Results for cell 203030003_R (from laboratory 1). (*B*) Results for cell 170518_4e (from laboratory 2).

A DBL implies a local, transient change of internal and external ion concentrations

A shift in the reversal potential of an ionic current implies a change in the ratio between an ion's internal and external concentration. From the changes in the reversal potential predicted by the model and the total initial concentrations of each ion, using the classic Nernst law at 35°C, we calculated the corresponding variation in the concentration inside and outside of a cell as a function of the input current. The results for the two neurons that were modeled are reported in Fig. 8 and suggest an approximately 20 mM change in (local) concentration for Na^+ and K^+ during a strong input. Because of the different initial concentrations on either side of the membrane for each ion, this change corresponds to approximately doubling the local concentration of internal Na^+ ions and a less than 15% decrease in the external Na^+. For K^+ ions, an approximate decrease of 15% of the internal concentration corresponded to a 10-fold increase in the external concentration. These results suggest that, during strong and sustained current injections, the local ion concentration of Na^+ and K^+ inside and outside of the membrane can change in a way that has not been considered previously when implementing computational models.

Response to synaptic inputs

The results discussed above were obtained under somatic current injection steps. Although this is by far the most studied experimental protocol for *in vitro* studies, neurons *in vivo* operate under a highly variable barrage of synaptic input activations. It was thus important to test what our model predicted under this condition. For this purpose, we investigated what would happen in a CA1 pyramidal cell during a bursting excitatory synaptic activity eliciting up and down states similar to those observed during spatial exploration (26) or expected from a theta-burst long term potentiation (LTP) induction protocol (27–30). A set of simulations ($n = 20$) was carried out, stimulating the model reproducing the traces from the 203030003_R cell with synaptic inputs targeting random locations of the distal and proximal dendrites to model excitatory EC and CA3 afferent pathways, respectively. To model a random synaptic background, all synapses were randomly (Poisson)

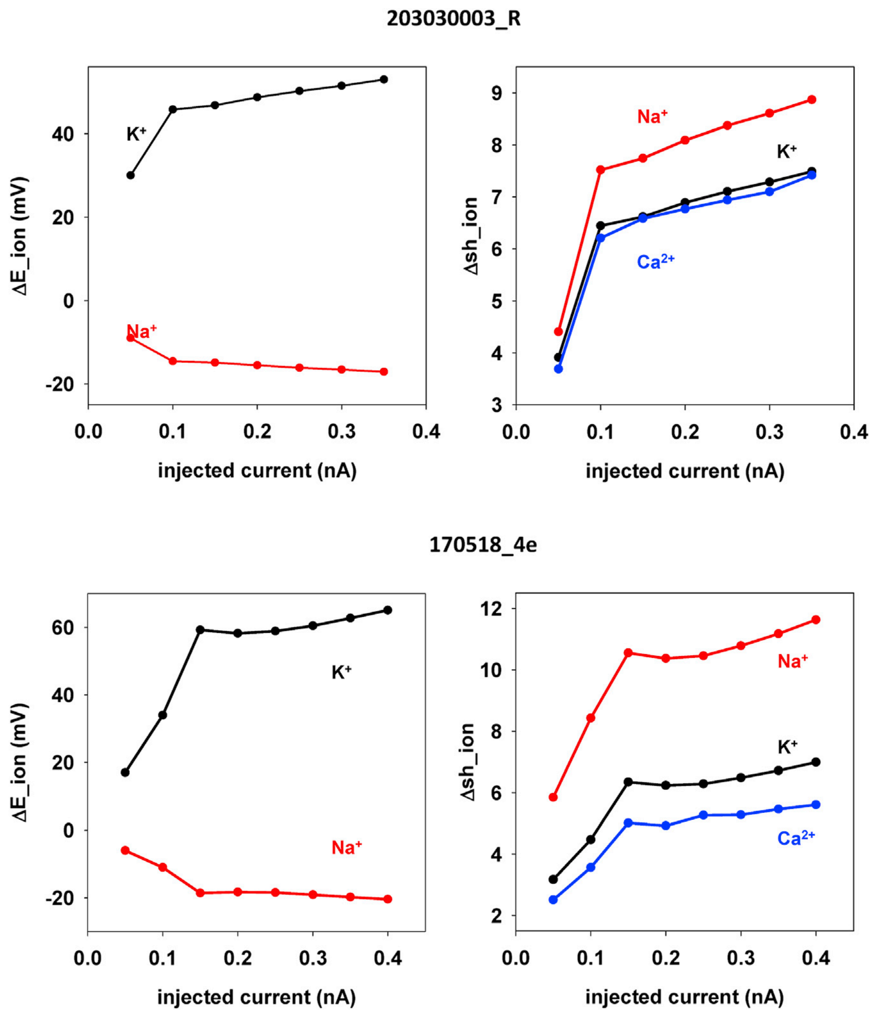

FIGURE 7 Shift of reversal potentials and channel kinetics. The plots show the shift of reversal potential with respect to control (left panels) and channel activation/inactivation kinetics (right panels) required to reproduce the DBL observed experimentally as a function of current injection. Top: results for cell 203030003_R (from laboratory 1). Bottom: results for cell 170518_4e (from laboratory 2).

activated at an average frequency of 4 Hz in the θ-rhythm range. SC synapses were further synchronously activated with bursts of 10 activations at 100 Hz elicited at an average frequency of 4 Hz. Examples of somatic and dendritic potential using the model with the mechanisms implementing the DBL (Fig. 9 *A*, blue traces) show that this stimulation protocol generates the typical up and down states observed in experimental recordings from hippocampus CA1 pyramidal neurons during spatial exploration (compare with Fig. 1 *g* in (16)). To illustrate the difference from the results obtained with a model not able to reproduce the DBL, we turned off the dynamic shifts by setting the parameter $B_j = 0$ (see Eq. [1] and Table 1) and adjusted the ionic peak conductances to obtain the same average number of APs. Fig. 9 *A*, bottom panels (black traces) illustrate the results under this condition. The neuron displayed roughly the same bursting activity but with much shorter interburst intervals and without the depolarizing envelope during the high-frequency synaptic activation periods. These effects were caused by the fixed Na^+ and K^+ current kinetic properties and reversal potentials. To study the possible consequences for somatic and dendritic signal processing, we calculated the average spectral power of the membrane potential during the 20 simulations at the soma and one dendritic location (Fig. 9 *B*, right panel). The power spectra (Fig. 9 *B*, black traces) show that the lack of DBL in a neuron model subjected to a theta-burst synaptic activation protocol will essentially filter out the signal component in the θ range (4–8 Hz) from the response and will amplify oscillations in the low γ range (∼30 Hz). These results suggest that the DBL mechanism can significantly affect a mouse hippocampus model operation during synaptic activity.

DISCUSSION

In this work we focused on a particular electrophysiological feature observed in somatic recordings from mice CA1 hippocampal pyramidal neurons: a progressive increase in the spike threshold and in the DBL observed in voltage traces during current injections. These effects have so far been ignored in conductance-based computational models, which

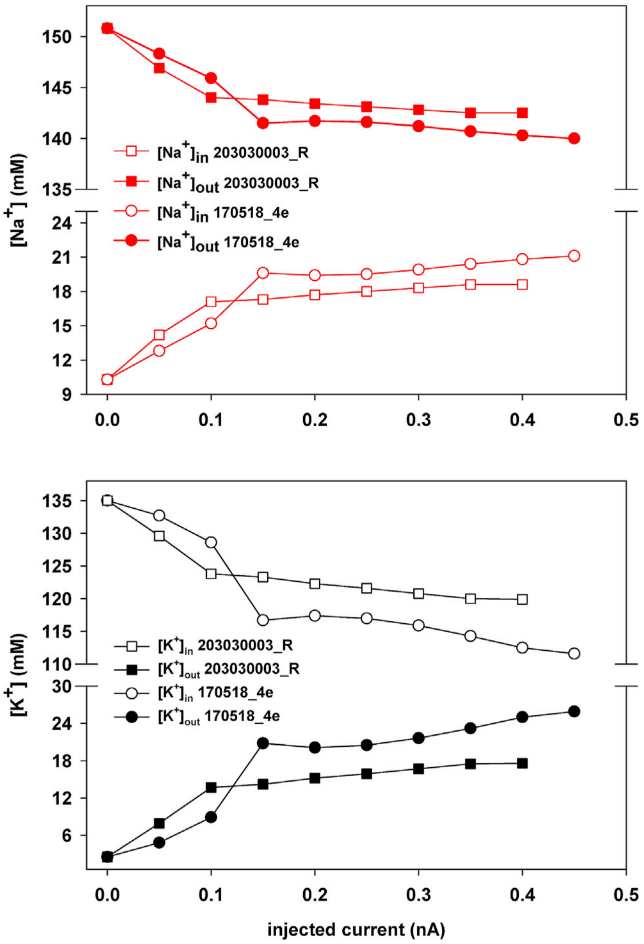

FIGURE 8 Local changes in the $[ion]_{in}/[ion]_{out}$ concentration ratio. Top: intra- and extracellular concentration of Na^+ ions, calculated from the reversal potential shift predicted for two neurons, as a function of the current injection. Bottom: same as in the top panel but for K^+ ions.

cannot reproduce them. We think that they may have important consequences for the input/output properties and dendritic signal processing of hippocampal neurons. In this work, we introduced empirical implementation of these mechanisms, which can be easily added to the current implementation of ion channel kinetics. The model suggested that the processes responsible for the DBL can be a local, stimulus-induced change in 1) the ionic microenvironment, resulting in a transient alteration of reversal potentials, and 2) in a change of membrane properties shifting channel kinetics, which may act as a sort of compensatory mechanism to restore full AP amplitude during a DBL.

The model suggested the physiologically plausible mechanisms that can be responsible for the substantial and dynamic change of the spike threshold and for a DBL up to more than 30 mV as a function of the external stimulus and without any significant effect on the spike amplitude. The predicted changes in the internal and external concentration ratios for Na^+ and K^+ (up to 10–20 mM) are consistent with experimental findings in rats during external stimulation of the cortical surface under physiological conditions (31,32), and with the results obtained with iono-phoretic applications of excitatory amino acids to the rat motor cortex (33). Interestingly, the changes predicted by our model are smaller than those measured in rats during hypoxia (50–100 mM (34)). In principle, ion-sensitive electrodes could be used to monitor large variations in pH, calcium, or potassium ions during agonist/antagonist applications, network oscillations, or epileptiform activities, as done in a few cases on hippocampal slices *ex vivo* (35,36). One study used these electrodes coupled with extracellular field potentials in hippocampal slices (37). However, to the best of our knowledge, ion-sensitive electrodes have not been adapted for use with single-cell patch-clamp analysis of excitability. Most likely, although highly sensitive, they do not have the capacity to sense single neuron-induced alterations in local ion flow.

Stimulus-induced changes in extracellular ion concentrations have been found and measured *in vivo* for more than 40 years; for example, in the adult cat sensorimotor cortex (31,32) and rat motor cortex (33). Furthermore, an increase in extracellular K^+ has also been correlated with pathological states like spreading depression and epilepsy (38,39) and with a change in the conformational state of membrane proteins that can modulate ionic channel currents (40). These are exactly the effects that we empirically modeled here in the context of reproducing the electrophysiological recordings obtained from mice. There are rather elegant mathematical implementations of ionic electrodiffusion (40,41) that, for example, can greatly help with understanding the complex mechanisms that can act to limit extracellular K^+ increases. In a more general theoretical context, previous papers on electrodiffusion have explicitly considered changes over time in reversal potentials (e.g., (42,43)) and their effect on membrane potential. However, they have not been validated against experimentally observed electrophysiological features. Finally, it may be argued that more sophisticated implementation of channel kinetics, such as Markov models (44), may be able to take into account the effects shown here. However, our results suggest that the transition rates between states of the channel are not only voltage dependent but also history dependent, implying that even a Markov model would be inadequate (45). It would be interesting to investigate these theories experimentally in more detail.

The stimulus- and history-dependent shifts in the ion channel kinetics may be caused by the direct effect of the (local) electric field generated by the input current. There are experimental and theoretical data suggesting that external or internal electric fields affect membrane permeability, among other properties (reviewed in (46)) and that polarization can alter the mechanocapacitive properties of a biological membrane (47). Our results suggest that the transition rates between states of the channel are not only voltage dependent but also history dependent. They support

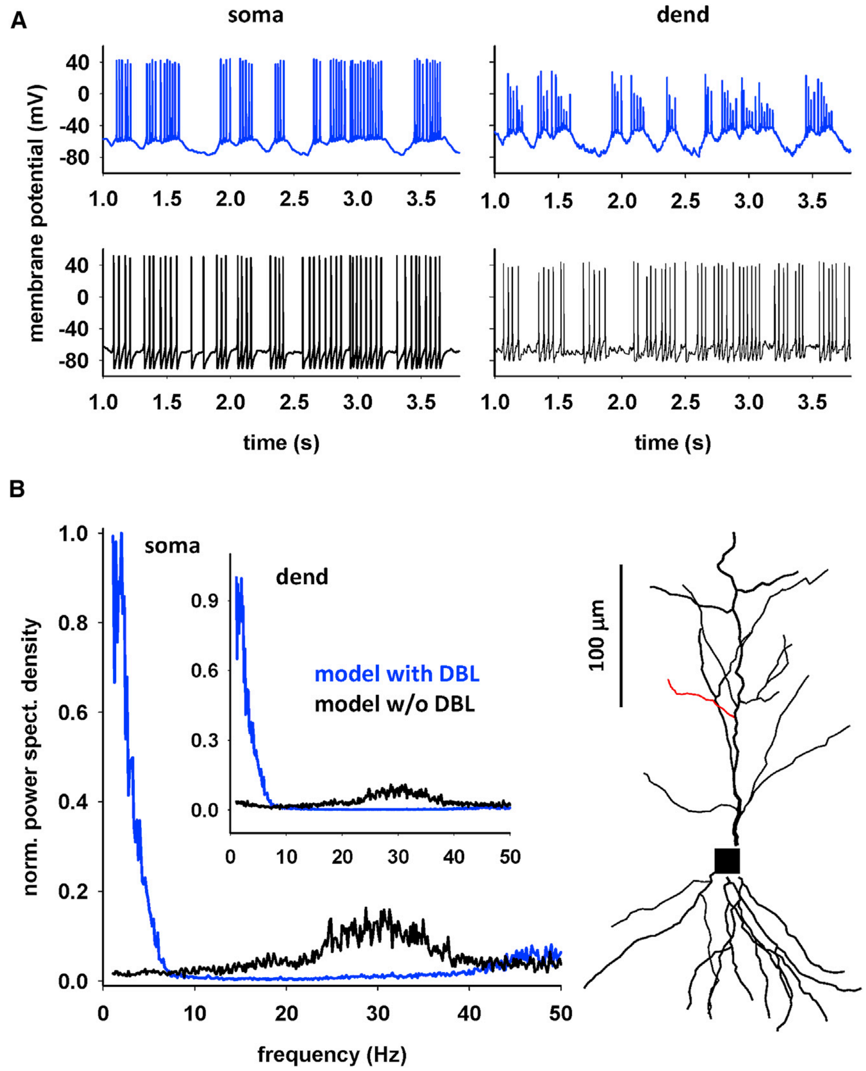

FIGURE 9 Response to synaptic inputs. (*A*) Somatic (left) and dendritic (right) membrane potential during an *in-vivo*-like synaptic input, using a model with DBL (blue traces) or a conventional model without DBL (black traces). (*B*) Normalized average power spectral density calculated from 20 10-s-long simulations; the image on the right shows the model neuron and the location of dendritic recording.

experimental findings suggesting that (at least) sodium channel properties, determined via voltage-clamp protocols, are history dependent (48).

Here we pointed out the possible mechanisms underlying these effects in a set of recordings from independent laboratories. This is the first explicit modeling demonstration of how to consider them in an empirical but biophysically plausible manner. Unfortunately, it is not known whether these effects can also be induced explicitly in rats. Stimulus-dependent effects on spike threshold have been observed in recordings from chick brain stem (49) and rat barrel cortex neurons (50). In general, it would be interesting to systematically explore the conditions under which they can be observed in other species. The results obtained here suggest that morphological differences and the consequent relative changes in the biophysical properties of the membrane and/or the size of the intra- and extracellular spatial domains relevant for ion fluxes can be important factors in determining the level of input that is needed to observe significant effects in different species. To the best of our knowledge, there are no experimental studies investigating in detail stimulus-induced dynamic changes of ion channel properties in hippocampal neurons. Part of the problem may be the technical difficulty of measuring a transient effect during a stimulus. Furthermore, the lack of significant changes in recordings from larger mammals, such as rats or humans, has also probably limited the interest in these effects. However, the model results point to potentially important consequences for I/O and dendritic integration properties that should be considered by modelers and experimentalists when they occur during normal *in vivo* activity. Modelers implementing detailed computational models of neurons and networks subjected to strong bursting inputs without taking into consideration the effects discussed here can miss low-frequency components (in the θ range in our case) of membrane oscillations at the soma and at dendritic synaptic locations. The former can alter the frequency content of the signal propagated to the rest of the

network, and the latter can unpredictably alter induction of local synaptic plasticity. Experimentalists may want to reconsider the range of input currents used *in vitro* to assess the intrinsic neurons' excitability. For example, at the same somatic current injection, hippocampal CA1 pyramidal neurons of mice fire APs at an average frequency that is approximately 5-fold higher than in rats (11). If this activity can be considered physiologically plausible, then it implies that the range of currents used to test neurons of other species needs to be extended to include strengths able to fire APs at the same rate as for the mouse. In this case, and assuming that the mammalian hippocampus has a similar local ionic microenvironment at rest, the model predicts that the same phenomena should also be observed for other species during inputs able to generate (somatic or dendritic) APs above approximately 40 Hz, with a DBL amount that will depend on the specific neuron properties.

SUPPORTING MATERIAL

Supporting material can be found online at https://doi.org/10.1016/j.bpj.2022.01.002.

AUTHOR CONTRIBUTIONS

D.B., R.M., and P.V. analyzed data and implemented the code. M.G., P.A.P., H.M., and V.L. performed the experiments. M.M. designed the study and wrote the manuscript with help from all other authors.

ACKNOWLEDGMENTS

This research has received funding from the European Union's Horizon 2020 Framework Program for Research and Innovation under Specific Grant Agreement 945539 (Human Brain Project SGA3) and Flag ERA JTC 2019 (MILEDI Project 825207), Fenix computing and storage resources under Specific Grant Agreement 800858 (Human Brain Project ICEI), and a grant from the Swiss National Supercomputing Centre (CSCS) under project ID ich002 and supported by the EU Joint Program–Neurodegenerative Disease Research (JPND) project CIRCPROT (jointly funded by BMBF, MIUR, ANR, and EU Horizon 2020 grant agreement no. 643417).

REFERENCES

1. Druckmann, S., T. K. Berger, …, I. Segev. 2011. Effective stimuli for constructing reliable neuron models. *PLoS Comput. Biol.* 7:e1002133. https://doi.org/10.1371/journal.pcbi.1002133.
2. Narayanan, R., and D. Johnston. 2007. Long-term potentiation in rat hippocampal neurons is accompanied by spatially widespread changes in intrinsic oscillatory dynamics and excitability. *Neuron.* 56:1061–1075. https://doi.org/10.1016/j.neuron.2007.10.033.
3. Narayanan, R., and D. Johnston. 2008. The h channel mediates location dependence and plasticity of intrinsic phase response in rat hippocampal neurons. *J. Neurosci.* 28:5846–5860. https://doi.org/10.1523/JNEUROSCI.0835-08.2008.
4. Marcelin, B., L. Chauvière, …, C. Bernard. 2009. h channel-dependent deficit of theta oscillation resonance and phase shift in temporal lobe epilepsy. *Neurobiol. Dis.* 33:436–447. https://doi.org/10.1016/j.nbd.2008.11.019.
5. Migliore, M., D. A. Hoffman, …, D. Johnston. 1999. Role of an A-type K+ conductance in the back-propagation of action potentials in the dendrites of hippocampal pyramidal neurons. *J. Comput. Neurosci.* 7:5–15. https://doi.org/10.1023/A:1008906225285.
6. Migliore, M., F. Cavarretta, …, G. M. Shepherd. 2015. Synaptic clusters function as odor operators in the olfactory bulb. *Proc. Natl. Acad. Sci. U S A.* 112:8499–8504. https://doi.org/10.1073/pnas.1502513112.
7. Enrico, P., M. Migliore, …, M. Diana. 2016. Morphofunctional alterations in ventral tegmental area dopamine neurons in acute and prolonged opiates withdrawal. A computational perspective. *Neuroscience.* 322:195–207. https://doi.org/10.1016/j.neuroscience.2016.02.006.
8. Carnevale, N. T., and M. L. Hines. 2006. The NEURON Book. Cambridge University Press.
9. Hines, M. L., A. P. Davison, and E. Muller. 2009. NEURON and Python. *Front. Neuroinform.* 3:1. https://doi.org/10.3389/neuro.11.001.2009.
10. Sivagnanam, S., A. Majumdar, …, N. T. Carnevale. 2013. Introducing the neuroscience gateway. *In* IWSG, Volume 993 of CEUR Workshop Proceedings, CEUR-WS.org.
11. Migliore, R., C. A. Lupascu, …, M. Migliore. 2018. The physiological variability of channel density in hippocampal CA1 pyramidal cells and interneurons explored using a unified data-driven modeling workflow. *PLoS Comput. Biol.* 14:e1006423. https://doi.org/10.1371/journal.pcbi.1006423.
12. Bianchi, D., A. Marasco, …, M. Migliore. 2012. On the mechanisms underlying the depolarization block in the spiking dynamics of CA1 pyramidal neurons. *J. Comput. Neurosci.* 33:207–225. https://doi.org/10.1007/s10827-012-0383-y.
13. Magee, J. C., and D. Johnston. 1995. Characterization of single voltage-gated Na+ and Ca2+ channels in apical dendrites of rat CA1 pyramidal neurons. *J. Physiol.* 487:67–90. https://doi.org/10.1113/jphysiol.1995.sp020862.
14. Hoffman, D. A., and D. Johnston. 1999. Neuromodulation of dendritic action potentials. *J. Neurophysiol.* 81:408–411. https://doi.org/10.1152/jn.1999.81.1.408.
15. Magee, J. C. 1999. Dendritic Ih normalizes temporal summation in hippocampal CA1 neurons. *Nat. Neurosci.* 2:508–514. https://doi.org/10.1038/9158.
16. Bianchi, D., P. De Michele, …, M. Migliore. 2014. Effects of increasing CREB-dependent transcription on the storage and recall processes in a hippocampal CA1 microcircuit. *Hippocampus.* 24:165–177. https://doi.org/10.1002/hipo.22212.
17. Megias, M., Z. Emri, …, A. I. Gulyàs. 2001. Total number and distribution of inhibitory and excitatory synapses on hippocampal CA1 pyramidal cells. *Neuroscience.* 102:527–540. https://doi.org/10.1016/s0306-4522(00)00496-6.
18. Castelli, L., M. J. Nigro, and J. Magistretti. 2007. Analysis of resurgent sodium-current expression in rat parahippocampal cortices and hippocampal formation. *Brain Res.* 1163:44–55. https://doi.org/10.1016/j.brainres.2007.05.065.
19. French, C. R., P. Sah, …, P. W. Gage. 1990. A voltage-dependent persistent sodium current in mammalian hippocampal neurons. *J. Gen. Physiol.* 95:1139–1157. https://doi.org/10.1085/jgp.95.6.1139.
20. Uebachs, M., T. Opitz, …, H. Beck. 2010. Efficacy loss of the anticonvulsant carbamazepine in mice lacking sodium channel beta subunits via paradoxical effects on persistent sodium currents. *J. Neurosci.* 30:8489–8501.
21. Migliore, M., and R. Migliore. 2012. Know your current I(h): interaction with a shunting current explains the puzzling effects of its pharmacological or pathological modulations. *PLoS One.* 7:e36867. https://doi.org/10.1371/journal.pone.0036867.
22. Pring, M. J. 2002. Technical Analysis Explained, Fourth edition. McGraw-Hill.
23. Klinker, F. 2011. Exponential moving average versus moving exponential average. *Math. Semesterber.* 58:97–107. https://doi.org/10.1007/s00591-010-0080-8.

24. Lupascu, C. A., A. Morabito, …, M. Migliore. 2016. A general procedure to study subcellular models of transsynaptic signaling at inhibitory synapses. *Front. Neuroinform.* 10:23. https://doi.org/10.3389/fninf.2016.00023.

25. Lupascu, C. A., A. Morabito, …, M. Migliore. 2020. Computational modeling of inhibitory transsynaptic signaling in hippocampal and cortical neurons expressing intrabodies against gephyrin. *Front. Cell. Neurosci.* 14:173. https://doi.org/10.3389/fncel.2020.00173.

26. Bittner, K. C., C. Grienberger, …, J. C. Magee. 2015. Conjunctive input processing drives feature selectivity in hippocampal CA1 neurons. *Nat. Neurosci.* 18:1133–1142. https://doi.org/10.1038/nn.4062.

27. Bliss, T. V., and G. L. Collingridge. 1993. A synaptic model of memory: long-term potentiation in the hippocampus. *Nature.* 361:31–39. https://doi.org/10.1038/361031a0.

28. Capocchi, G., M. Zampolini, and J. Larson. 1992. Theta-burst stimulation is optimal for induction of LTP at both apical and basal dendritic synapses on hippocampal CA1 neurons. *Brain Res.* 591:332–336. https://doi.org/10.1016/0006-8993(92)91715-q.

29. Hernandez, R. V., M. M. Navarro, and R. G. LeBaron. 2005. Differences in the magnitude of long-term potentiation produced by q-burst and high frequency stimulation protocols matched in stimulus number. *Brain Res. Brain Res. Protoc.* 15:6–13. https://doi.org/10.1016/j.brainresprot.2005.02.003.

30. Migliore, M., G. De Simone, and R. Migliore. 2015. Effect of the initial synaptic state on the probability to induce long-term potentiation and depression. *Biophys. J.* 108:1038–1046. https://doi.org/10.1016/j.bpj.2014.12.048.

31. Dietzel, I., U. Heinemann, …, H. D. Lux. 1980. Transient changes in the size of the extracellular space in the sensorimotor cortex of cats in relation to stimulus-induced changes in potassium concentration. *Exp. Brain Res.* 40:432–439. https://doi.org/10.1007/BF00236151.

32. Dietzel, I., U. Heinemann, …, H. D. Lux. 1982. Stimulus-induced changes in extracellular Na+ and Cl- concentration in relation to changes in the size of the extracellular space. *Exp. Brain Res.* 46:73–84. https://doi.org/10.1007/BF00238100.

33. Pumain, R., and U. Heinemann. 1985. Stimulus- and amino acid-induced calcium and potassium changes in rat neocortex. *J. Neurophysiol.* 53:1–16. https://doi.org/10.1152/jn.1985.53.1.1.

34. Müller, M., and G. G. Somjen. 2000. Na(+) and K(+) concentrations, extra- and intracellular voltages, and the effect of TTX in hypoxic rat hippocampal slices. *J. Neurophysiol.* 83:735–745. https://doi.org/10.1152/jn.2000.83.2.735.

35. LeBeau, F. E. N., S. K. Towers, …, E. H. Buhl. 2002. Fast network oscillations induced by potassium transients in the rat hippocampus in vitro. *J. Physiol.* 542:167–179. https://doi.org/10.1113/jphysiol.2002.015933.

36. Haack, N., S. Durry, …, R. Rose. 2015. Double-barreled and concentric microelectrodes for measurement of extracellular ion signals in brain tissue. *J. Vis. Exp.* 53058. https://doi.org/10.3791/53058.

37. Fedirko, N., N. Svichar, and M. Chesler. 2006. Fabrication and use of high-speed, concentric H+- and Ca2+-selective microelectrodes suitable for in vitro extracellular recording. *J. Neurophysiol.* 96:919–924. https://doi.org/10.1152/jn.00258.2006.

38. Kager, H., W. J. Wadman, and G. G. Somjen. 2000. Simulated seizures and spreading depression in a neuron model incorporating interstitial space and ion concentrations. *J. Neurophysiol.* 84:495–512. https://doi.org/10.1152/jn.2000.84.1.495.

39. Somjen, G. G. 2001. Mechanisms of spreading depression and hypoxic spreading depression-like depolarization. *Physiol. Rev.* 81:1065–1096. https://doi.org/10.1152/physrev.2001.81.3.1065.

40. Halnes, G., T. MaÈki-Marttunen, …, G. T. Einevoll. 2016. Effect of ionic diffusion on extracellular potentials in neural tissue. *PLoS Comput. Biol.* 12:e1005193. https://doi.org/10.1371/journal.pcbi.1005193.

41. Ellingsrud, A. J., A. Solbrå, …, M. E. Rognes. 2020. Finite element simulation of ionic electrodiffusion in cellular geometries. *Front. Neuroinform.* 14:11. https://doi.org/10.3389/fninf.2020.00011.

42. Nygren, A., and J. A. Halter. 1999. A general approach to modeling conduction and concentration dynamics in excitable cells of concentric cylindrical geometry. *J. Theor. Biol.* 199:329–358. https://doi.org/10.1006/jtbi.1999.0962.

43. Sætra, M. J., G. T. Einevoll, and G. Halnes. 2020. An electrodiffusive, ion conserving Pinsky-Rinzel model with omeostatic mechanisms. *PLoS Comput. Biol.* 16:e1007661. https://doi.org/10.1371/journal.pcbi.1007661.

44. Navarro, M. A., A. Salari, …, L. S. Milescu. 2020. Sodium channels implement a molecular leaky integrator that detects action potentials and regulates neuronal firing. *Elife.* 9:e54940. https://doi.org/10.7554/eLife.54940.

45. Silva, M. P., C. G. Rodrigues, …, R. A. Nogueira. 2021. Memory in ion channel kinetics. *Acta Biotheor.* https://doi.org/10.1007/s10441-021-09415-1.

46. Galassi, V. V., and N. Wilke. 2021. On the coupling between mechanical properties and electrostatics in biological membranes. *Membranes.* 11:478. https://doi.org/10.3390/membranes11070478.

47. Mosgaard, L. D., K. A. Zecchi, and T. Heimburg. 2015. Mechano-capacitive properties of polarized membranes. *Soft Matter.* 11:7899–7910. https://doi.org/10.1039/C5SM01519G.

48. Majumdar, S., and S. K. Sikdar. 2007. Periodicity in Na+ channel properties alters excitability of a model neuron. *Biochem. Biophys. Res. Commun.* 359:908–914. https://doi.org/10.1016/j.bbrc.2007.05.185.

49. Lubejko, S. T., B. Fontaine, …, K. M. MacLeod. 2019. Spike threshold adaptation diversifies neuronal operating modes in the auditory brain stem. *J. Neurophysiol.* 122:2576–2590. https://doi.org/10.1152/jn.00234.2019.

50. Wilent, W. B., and D. Contreras. 2015. Stimulus-dependent changes in spike threshold enhance feature selectivity in rat barrel cortex neurons. *J. Neurosci.* 25:2983–2991. https://doi.org/10.1523/JNEUROSCI.4906-04.2005.

Biophysical Journal, Volume *121*

Supplemental information

Membrane electrical properties of mouse hippocampal CA1 pyramidal neurons during strong inputs

Daniela Bianchi, Rosanna Migliore, Paola Vitale, Machhindra Garad, Paula A. Pousinha, Helene Marie, Volkmar Lessmann, and Michele Migliore

Supporting Material

Membrane electrical properties of mouse hippocampal CA1 pyramidal neurons during strong inputs

D. Bianchi[1†], R. Migliore[1†], P. Vitale[1], M. Garad[2], P.A. Pousinha[3], H. Marie[3], V. Lessmann[2], M. Migliore[1*]

[1]Institute of Biophysics, National Research Council, Palermo, Italy

[2]Otto-von-Guericke University, Magdeburg, Germany

[3]Université Côte d'Azur, CNRS, IPMC, France

1. The Na^+ channel's model

In this section we describe in detail the kinetic of the transient Na^+ channel, used in this paper to reproduce the experimental findings, and implemented assuming that stimulus amplitude and duration generate a shift of the kinetics and reversal potential.

We started from a HH-like implementation of channel kinetics. The sodium current i_{Na} was defined as

$$i_{Na}(t,v) = G_{Na}(t,v) \cdot (v - E_{Na}(t,v)),$$

where v is the membrane potential, E_{Na} the reversal potential, and $G_{Na}(t,v) = g_{Na} \cdot m^3(t,v) \cdot h(t,v)$ describes the channel's conductance change. It depends on the peak conductance g_{Na} and on the activation and inactivation variables $m(t,v)$ and $h(t,v)$, respectively.

In our model, the reversal potential and the shift of activation/inactivation kinetics, with respect to their original values, were modulated by the EMA's function $f_{vrun}(t,v)$ (see main text for its definition) as

$$E_{Na}(t,v) = E_{Na}(t=0) - \alpha_{ENa} \cdot f_{vrun}(t,v)$$

$$sh_{Na}(t,v) = sh_{Na}(t=0) - \alpha_{Na} \cdot f_{vrun}(t,v)$$

Following this modulation, the original equations for the activation and inactivation variables

$$\frac{dm(t,v)}{dt} = \frac{m_\infty(t,v) - m(t,v)}{\tau_m(t,v)}$$

$$\frac{dh(t,v)}{dt} = \frac{h_\infty(t,v) - \mathrm{h}(t,v)}{\tau_h(t,v)}$$

were modified as follows.

The functions $a_m(t,v)$ and $b_m(t,v)$, defining the activation gate variable

$$m_\infty(t,v) = \frac{a_m(t,v)}{a_m(t,v) + b_m(t,v)}$$

and its time constant

$$\tau_m(t,v) = \begin{cases} \dfrac{2}{a_m(t,v) + b_m(t,v)} & \text{if} \quad \dfrac{2}{a_m(t,v) + b_m(t,v)} > 0.02 \\ \quad else & 0.02\ ms \end{cases}$$

were modified as

$$a_m(t,v) = \begin{cases} \dfrac{0.4 \cdot \left[v - \left(v_m^{1/2} + sh_{Na}(t,v)\right)\right]}{1 - e^{-\frac{v - \left(v_m^{1/2} + sh_{Na}(t,v)\right)}{qa}}} & if\ \left|v - v_m^{1/2}\right| > 10^{-6} mV \\ \quad else & 0.4 \cdot qa \end{cases}$$

$$b_m(t,v) = \begin{cases} \dfrac{-0.124 \cdot \left[v - \left(v_m^{1/2} + sh_{Na}(t,v)\right)\right]}{1 - e^{\frac{v - \left(v_m^{1/2} + sh_{Na}(t,v)\right)}{qa}}} & if\ \left|v - v_m^{1/2}\right| > 10^{-6} mV \\ \quad else & 0.124 \cdot qa \end{cases}$$

Similarly, for the inactivation gate variable

$$h_\infty(t,v) = \frac{1}{\left(1 + e^{(v - v_h^{1/2} - sh_{Na}(t,v))/qinf)}\right)}$$

$$\tau_h(t,v) = \begin{cases} \dfrac{2}{a_h(t,v) + b_h(t,v)} & \text{if} \quad \dfrac{2}{a_h(t,v) + b_h(t,v)} > 0.05 \\ \quad else & 0.05\ ms \end{cases}$$

The functions were modified as

$$a_h(t,v) = \begin{cases} \dfrac{0.03 \cdot \left[v - \left(v_h^{1/2} + sh_{Na}(t,v)\right)\right]}{1 - e^{-\frac{v - \left(v_h^{1/2} + sh_{Na}(t,v)\right)}{qd}}} & if\ \left|v - v_h^{1/2}\right| > 10^{-6} mV \\ \quad else & 0.03 \cdot qd \end{cases}$$

$$b_h(t,v) = \begin{cases} \dfrac{-0.01 \cdot \left[v - \left(v_h^{\frac{1}{2}} + sh_{Na}(t,v)\right)\right]}{1 - e^{\frac{v - \left(v_h^{\frac{1}{2}} + sh_{Na}(t,v)\right)}{qg}}} & if\ \left|v - v_h^{\frac{1}{2}}\right| > 10^{-6} mV \\ \quad else & 0.01 \cdot qg \end{cases}$$

with $qa = 7.2\ mV$, $v_m^{1/2} = -27\ mV$, $qinf = 2\ mV$, $v_h^{1/2} = -45\ mV$, $qd = 1.5\ mV$, $qg = 1.5\ mV$.

An analogous implementation of the dynamic shift was applied to potassium and calcium ionic currents.

2. The persistent Na^+ channel model

In this section we explicitly describe the equations governing the Na^+ persistent current used for Fig.5. The sodium current i_{NaP} was defined as

$$i_{NaP}(t,v) = G_{NaP}(t,v) \cdot (v - E_{Na}),$$

where

$$G_{NaP}(t,v) = g_{NaP} \cdot m(t,v) \cdot h(t,v),$$

E_{Na} is the reversal potential, g_{NaP} the peak conductance, and $m(t,v)$ and $h(t,v)$ the activation and inactivation variables, described by the equations:

$$\frac{dm(t,v)}{dt} = \frac{m_\infty(t,v) - m(t,v)}{\tau_m}$$

and

$$\frac{dh(t,v)}{dt} = \frac{h_\infty(t,v) - \mathrm{h}(t,v)}{\tau_h}$$

where

$$m_\infty(t,v) = \frac{1}{[1 + \exp(-(v + 52.3)/6.8)]}$$

$$h_\infty(t,v) = \frac{1}{[1 + \exp((v + 48)/10)]}$$

with $\tau_h = 4000\ ms$, and $\tau_m = 1$ if $(m < m_\infty)$, else $\tau_m = 300$.

Simulation and model files are available for download at http://senselab.med.yale.edu/ModelDB/ acc.n. 266900.
